# Synthesis, Crystal Structure and Luminescent Property of Cd (II) Complex with *N*-Benzenesulphonyl-*L*-leucine

**DOI:** 10.3390/ma5091626

**Published:** 2012-09-14

**Authors:** Xishi Tai, Jinhe Jiang

**Affiliations:** College of Chemistry and Chemical Engineering, Weifang University, Weifang 261061, China; E-Mail: jiangjinhe2006@163.com (J.H.)

**Keywords:** *N*-Benzenesulphonyl-*L*-leucine, trinuclear Cd (II) complex, synthesis, crystal structure, luminescent property

## Abstract

A new trinuclear Cd (II) complex [Cd_3_(L)_6_(2,2-bipyridine)_3_] [L = *N*-phenylsulfonyl-*L*-leucinato] has been synthesized and characterized by elemental analysis, IR and X-ray single crystal diffraction analysis. The results show that the complex belongs to the orthorhombic, space group *P*2_1_2_1_2_1_ with *a* = 16.877(3) Å, *b* = 22.875(5) Å, *c* = 29.495(6) Å, *α* = *β* = *γ* = 90°, *V* = 11387(4) Å^3^, *Z* = 4, *D_c_*= 1.416 μg·m^−3^, *μ* = 0.737 mm^−1^, *F* (000) = 4992, and final *R*_1_ = 0.0390, *ωR*_2_ = 0.0989. The complex comprises two seven-coordinated Cd (II) atoms, with a N_2_O_5_ distorted pengonal bipyramidal coordination environment and a six-coordinated Cd (II) atom, with a N_2_O_4_ distorted octahedral coordination environment. The molecules form one dimensional chain structure by the interaction of bridged carboxylato groups, hydrogen bonds and π-π interaction of 2,2-bipyridine. The luminescent properties of the Cd (II) complex and *N*-Benzenesulphonyl-*L*-leucine in solid and in CH_3_OH solution also have been investigated.

## 1. Introduction

Investigation of the inorganic-organic hybrid materials with carboxylate ligands has gained considerable attention during the last decade due to their attractive structures and promising potential applications for catalysis, gas storage, magnetic, luminescence materials [1,2,3,4,5,6,7]. Structural studies have shown that the organic ligands containing multi-oxygen and nitrogen atoms can coordinate with metal ions in different ways, resulting in the formation of various metal-organic frameworks with specific topologies and useful properties. The Cd (II) complexes have gained considerable attention due to their luminescent properties [4]. We have been exploring the preparation of inorganic-organic hybrid materials by combining metal ions and organic ligands containing multi-oxygen and nitrogen atoms. We have now synthesized a new hybrid material, [Cd_3_(L)_6_(2,2-bipyridine)_3_] [L= *N*-phenylsulfonyl-*L*-leucinato]. The luminescent properties of the Cd (II) complex and *N*-Benzenesulphonyl-*L*-leucine in solid and in CH_3_OH solution also have been investigated.

## 2. Results and Discussion

### 2.1. IR Spectra

The *ν*_as_ (COOH), *ν*_s_ (COOH) and *ν* (C=N) vibrations of free ligand are at 1,659 cm^−1^ and 1,436 cm^−1^ and 1587 cm^−1^, respectively. For the Cd (II) complex, they shift to 1,587 cm^−1^, 1,402 cm^−1^ and 1552 cm^−1^, respectively, which suggest that the oxygen atoms of COO^-^ and the nitrogen atoms of 2,2-bipyridine coordinate to Cd (II) ions [8]. The difference between the *ν*_as_ (COO^−^) and *ν*_s_ (COO^−^) band is 185 cm^−1^, indicating a bidentate carboxylate moiety, consistent with the X-ray structural analysis. The new band at 419 cm^−1^ is assigned to the *ν* (Cd-O) vibration.

### 2.2. Structure Description

**Figure 1:**
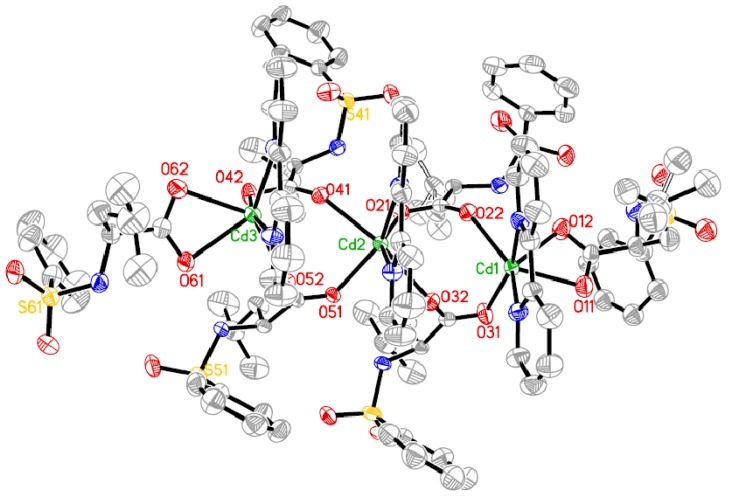
The molecular structure of the Cd (II) complex.

**Figure 2:**
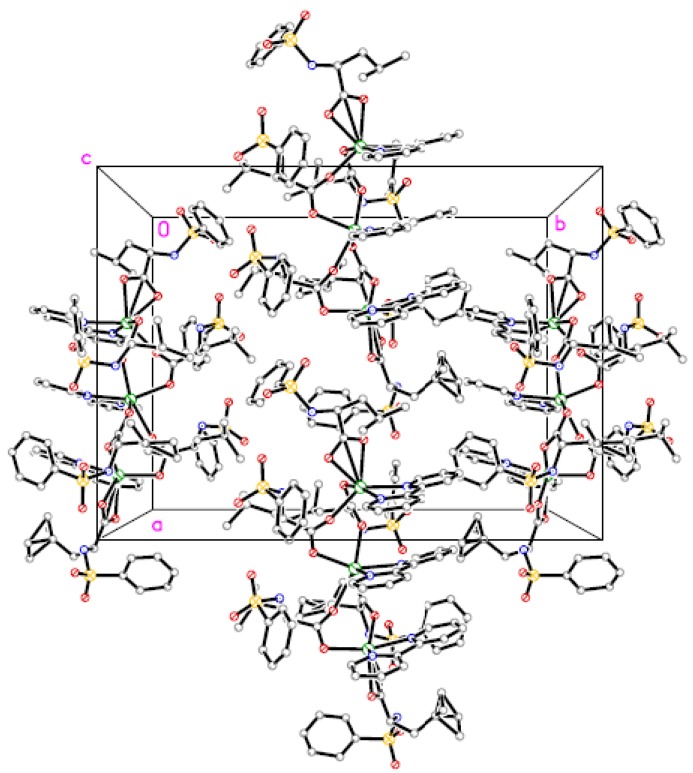
The molecular packing arrangement of the Cd (II) complex.

**Figure 3:**
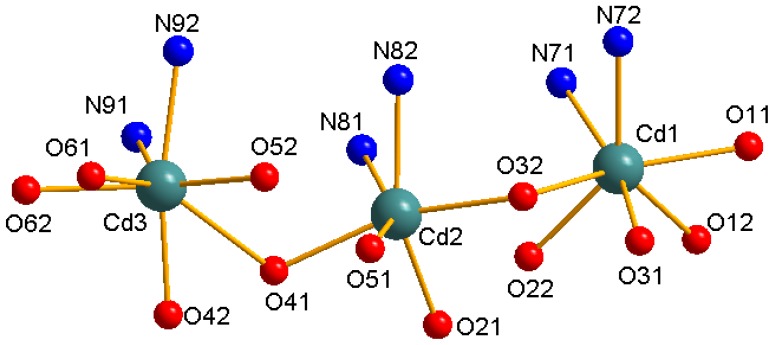
The coordination environment of the Cd (II) ions.

**Figure 4:**
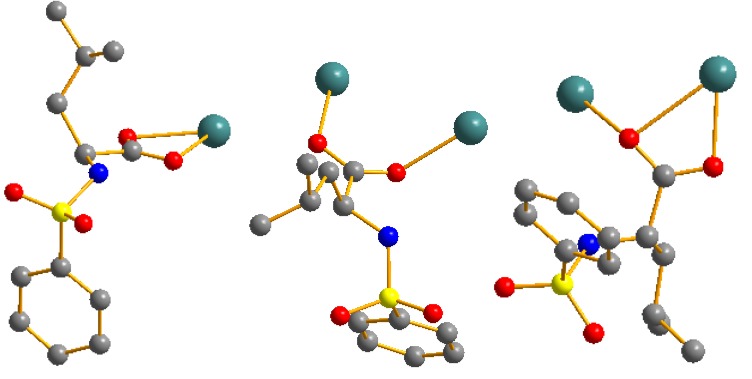
The coordination options of carboxylates in the Cd (II) complex.

The molecular structure and molecular packing arrangement are shown in Figure 1 and Figure 2, respectively. Figure 3 shows the coordination environment of the Cd (II) atom. From Figure 1 and Figure 3, we can see that two coordination environments of the Cd (II) atoms exist in the complex. The first coordination environment of the Cd (II) atom [Cd1, Cd3] is a distorted pengonal bipyramidal coordination environment with five oxygen atoms from the *N*-phenylsulfonyl-*L*-leucinato ligand, two nitrogen atoms from 2,2-bipyridine. The second coordination environment of the Cd (II) atom [Cd2] is a distorted octahedral coordination environment with four oxygen atoms from the *N*-phenylsulfonyl-*L*-leucinato ligand, two nitrogen atoms from 2,2-bipyridine. The carboxylates have three coordination ways of in the Cd (II) complex (Figure 4). The distances of the Cd-O bonds and Cd-N bonds are in the range of 2.244(3)–2.450(4) Å and 2.329(4)–2.366(4) Å, respectively. The aromatic rings of 2,2-bipyridine in the complex are nearly parallel, the dihedral angle and distance between ring 1 (containing N71 and N72) and ring 2 (containing N81 and N82) are 7.8(3)° and 3.846(4) Å, ring 2 (containing N81 and N82) and ring 3 (containing N91 and N92) are 6.2(3)° and 3.886(4) Å. The hydrogen bonds and π-π interaction of 2,2-bipyridine rings help to stabilize the structure. The bond lengths of Cd-O are similar to the Cd-O bond lengths reported previously [9,10]. The aromatic rings in the molecules do not show any unusual features, and the bond lengths and bond angles are within the range of normal values.

### 2.3. Luminescent Properties

**Figure 5:**
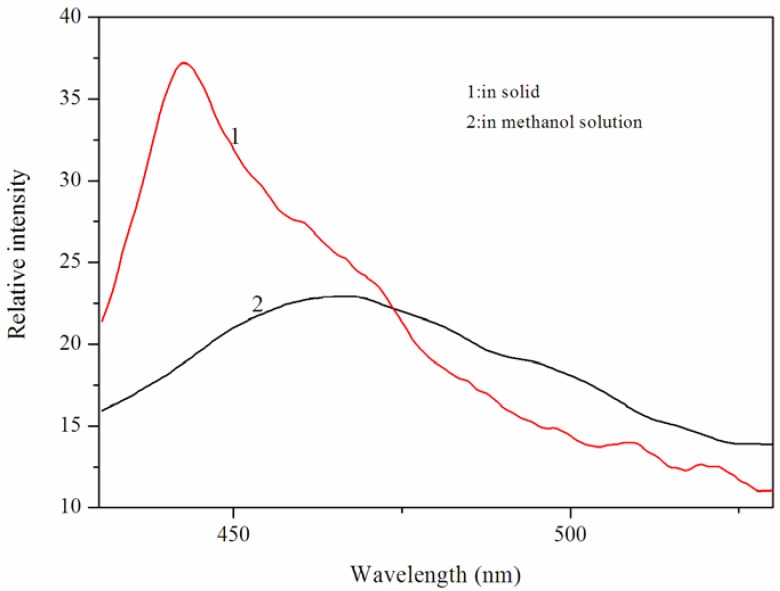
The emission spectrum of the Cd (II) complex. The excitation and emission slit widths were 5 nm.

Previous studies have shown that the Cd (II) complexes exhibit luminescent properties [11]. Hence, we investigated the luminescent properties of the Cd (II) complex in the solid-state and in CH_3_OH solution (1.06 × 10^−5^ mol L^−1^) at room temperature. The emission spectra of the Cd (II) complex are shown in Figure 5. In the solid state, the Cd (II) complex displays strong luminescent emission bands at 442 nm when excited at 335 nm. And in CH_3_OH solution, the Cd (II) complex displays strong luminescent emission bands at 457 nm when excited at 335 nm. For excitation wavelengths between 280 and 420 nm, there is no obvious emission observed for the *N*-benzenesulphonyl-*L*-leucine ligand. Compared with the emission maximum of the Cd (II) complex in methanol solution, the emission maximum of the Cd (II) complex in solid was blue shifted, which may be due to the quenching effect of the methanol solvent.

## 3. Experimental Section

### 3.1. Materials and Methods

The *N*-benzenesulphonyl-*L*-leucine ligand was prepared according to the method reported in the literature [5]. Other chemicals were of reagent grade and were used without further purification.

Carbon, hydrogen and nitrogen analyses were obtained using an Elementar Vario III EL elemental analyzer. Infrared spectra were recorded on a Nicolet AVATAR 360 FTIR spectrophotometer with KBr in the range of 400 cm^−1^–4000 cm^−1^. Mass spectrum was performed on a VG ZAB-HS Fast-atom bombardment (FAB) instrument. Excitation and emission spectra were obtained on a PE LS-55 spectrometer at room temperature. X-ray diffraction data of the Cd (II) complex was collected on a Bruker smart CCD diffractometer.

### 3.2. Synthesis of Cd (II) Complex

A methanol solution of 0.5 m mol (0.1543 g) cadmium nitrate tetrahydrate was added to a solution containing 1.0 m mol (0.2710 g) of *N*-benzenesulphonyl-*L*-leucine and 1.0 m mol (0.04 g) of sodium hydroxide in 10 mL CH_3_OH. The mixture was continuously stirred for 2 h at refluxing temperature. The mixture was cooled at room temperature, and was collected by filtration. By evaporation in air at room temperature, a single crystal suitable for X-ray determination was obtained from methanol solution after 15 days. Elementary analysis: calcd for C_102_H_120_Cd_3_N_12_O_24_S_6_: C, 50.42; H, 4.94; N, 6.92%; found: C, 50.77; H, 4.58; N, 6.73%. IR *ν*_max_ (cm^−1^): *ν*_as_ (COO^−^):1,587 cm^−1^, *ν*_s_ (COO^−^):1402 cm^−1^, *ν* (-SO_2_-NH-): 3,249 cm^−1^, 1,320 cm^−1^,1,156 cm^−1^, *ν* (C=N): 1,552 cm^−1^, *ν* (Cd-O):419 cm^−1^.

### 3.3. X-Ray Crystallography

Single crystal X-ray diffraction data were collected on a Bruker smart CCD diffractometer at 153(2) K using graphite-monochromatic Mo *Kα* radiation (*λ* = 0.71073 Å). The structure was solved by the direct method and refined with full-matrix least-squares techniques using SHELXL-97 [12]. All non-hydrogen atoms were refined anisotropically, and all hydrogen atoms were put in calculated positions. Molecular graphics were drawn with the program package SHELXTL-97 crystallographic software package [13]. The main crystal data of the collection and refinement details of the Cd (II) complex are summarized in Table 1. Selected bond lengths and angles are listed in Table 2.

**Table 1:** Crystallographic data for the Cd (II) complex.

Crystallographic parameter	Crystallographic data
Formula	C_102_H_120_Cd_3_N_12_O_24_S_6_
Formula weight	2427.66
Crystal system	Orthorhombic
Space group	*P*2_1_2_1_2_1_
*a*(Å)	16.877(3)
*b*(Å)	22.875(5)
*c*(Å)	29.495(6)
Z	4
*F*(000)	4992
Temperature (K)	153(2)
*V* (Å ^3^)	11387(4)
Calculated density (μg·m^−3^)	1.416
Crystal size (mm^3^)	0.24 × 0.20 × 0.12
*μ* (mm^−1^)	0.737
*S*	1.060
Limiting indices	−20≤ *h* ≤ 20, −26 ≤ *k* ≤ 27, −35 ≤ *l* ≤ 34
Reflections collected/unique	20023/17894
*R*_1_, *wR*_2_ [all data]	0.0452, 0.1029
*R*_1_, *wR*_2_ [*I* > 2*σ*(*I*)]	0.0390, 0.0989
Largest diff.peak and hole (e·Å^−3^)	1.213–1.250

**Table 2:** Selected bond lengths (Å) and angles (°) for the Cd (II) complex.

Bonds	Bond parameter	Bonds	Bond parameter
Cd1-O31	2.241(3)	Cd2-O41	2.386(3)
Cd1-N71	2.329(4)	Cd2-O32	2.408(3)
Cd1-O22	2.331(3)	Cd3-O42	2.244(3)
Cd1-O11	2.331(4)	Cd3-O52	2.310(3)
Cd1-N72	2.366(4)	Cd3-N91	2.330(4)
Cd1-O12	2.450(4)	Cd3-N92	2.347(5)
Cd2-O21	2.253(3)	Cd3-O62	2.360(4)
Cd2-O51	2.263(3)	Cd3-O61	2.420(4)
Cd2-N81	2.335(4)	-	-
Cd2-N82	2.341(4)	-	-
O31-Cd1-N71	161.91(14)	O41-Cd2-N81	78.30(13)
O31-Cd1-O22	92.09(14)	O41-Cd2-N82	116.50(13)
N71-Cd1-O22	84.39(14)	O21-Cd2-O32	89.06(12)
O31-Cd1-O11	94.74(16)	O51-Cd2-O32	81.05(12)
N71-Cd1-O11	100.64(17)	N81-Cd2-O32	120.38(13)
O11-Cd1-O22	133.05(13)	O32-Cd2-N82	78.95(13)
O31-Cd1-N72	102.03(15)	O32-Cd2-O41	160.16(12)
N71-Cd1-N72	70.04(15)	O42-Cd3-O52	94.83(13)
N72-Cd1-O22	137.92(14)	N91-Cd3-O42	106.22(15)
O11-Cd1-N72	85.50(15)	N91-Cd3-O52	133.77(15)
O31-Cd1-O12	99.51(15)	O42-Cd3-N92	168.28(15)
N71-Cd1-O12	97.17(16)	N92-Cd3-O52	80.53(14)
O12-Cd1-O22	78.55(13)	N91-Cd3-N92	70.57(16)
O11-Cd1-O12	54.50(14)	O42-Cd3-O62	91.90(15)
N72-Cd1-O12	135.80(15)	O52-Cd3-O62	135.51(13)
O21-Cd2-O51	103.84(12)	N91-Cd3-O62	85.27(16)
O21-Cd2-N81	97.39(14)	O62-Cd3-N92	98.95(16)
O51-Cd2-N81	150.20(14)	O61-Cd3-O42	100.38(13)
O21-Cd2-N82	153.49(14)	O61-Cd3-O52	81.03(12)
O51-Cd2-N82	97.58(13)	N91-Cd3-O61	132.21(14)
N81-Cd2-N82	69.56(14)	O61-Cd3-N92	89.58(15)
O21-Cd2-O41	81.37(11)	O62-Cd3-O61	54.52(13)
O51-Cd2-O41	84.41(12)	-	-

## 4. Conclusions

In summary, by selecting the Cd (II) ion as a knot, and *N*-phenylsulfonyl-*L*-leucinato and 2,2-bipyridine as a building block, a new complex [Cd_3_(L)_6_(2,2-bipyridine)_3_] has been synthesized and structurally characterized. The complex comprises two seven-coordinated Cd (II) atoms, with a N_2_O_5_ distorted pengonal bipyramidal coordination environment and a six-coordinated Cd (II) atom, with a N_2_O_4_ distorted octahedral coordination environment. The molecules form a one dimensional chain structure by the interaction of bridged carboxylato groups and π-π interaction of 2,2-bipyridine. The Cd (II) complex exhibited fluorescence in solid and in CH_3_OH. Based on those results, a series of new Cd (II) complex could be designed and synthesized to optimize the luminescent properties.

## Supplementary Material

Crystallographic data for the structure reported in this paper has been deposited with the Cambridge Crystallographic Data Centre as supplementary publication No.CCDC 890837. Copy of the data can be obtained free of charge on application to CCDC, 12 Union Road, Cambridge CB2 1EZ, UK (Fax: +44-1223-336-033; E-Mail: deposit@ccdc.cam.ac.uk).
